# Proteomics studies confirm the presence of alternative protein isoforms on a large scale

**DOI:** 10.1186/gb-2008-9-11-r162

**Published:** 2008-11-18

**Authors:** Michael L Tress, Bernd Bodenmiller, Ruedi Aebersold, Alfonso Valencia

**Affiliations:** 1 Structural Biology and Biocomputing Programme, Spanish National Cancer Research Centre (CNIO), C. Melchor Fernandez Almagro, Madrid 28029, Spain; 2 Institute of Molecular Systems Biology, ETH, Wolfgang-Pauli-Str., 8093 Zurich, Switzerland; 3 Institute for Systems Biology, Seattle, WA 98103, USA; 4 Competence Center for Systems Physiology and Metabolic Diseases, ETH Zurich, 8093 Zurich, Switzerland; 5 Faculty of Science, University of Zurich, 8057 Zurich, Switzerland

## Abstract

**Background:**

Alternative splicing of messenger RNA permits the formation of a wide range of mature RNA transcripts and has the potential to generate a diverse spectrum of functional proteins. Although there is extensive evidence for large scale alternative splicing at the transcript level, there have been no comparable studies demonstrating the existence of alternatively spliced protein isoforms.

**Results:**

Recent advances in proteomics technology have allowed us to carry out a comprehensive identification of protein isoforms in *Drosophila*. The analysis of this proteomic data confirmed the presence of multiple alternative gene products for over a hundred *Drosophila *genes.

**Conclusions:**

We demonstrate that proteomics techniques can detect the expression of stable alternative splice isoforms on a genome-wide scale. Many of these alternative isoforms are likely to have regions that are disordered in solution, and specific proteomics methodologies may be required to identify these peptides.

## Abstract 2

Stably expressed alternatively-spliced protein isoforms are produced on a genome-wide scale in Drosophila.

## Background

The alternative splicing of pre-messenger RNA (mRNA) allows for the generation of diverse mature RNA transcripts from a single mRNA strand [1,2]. Recent studies have estimated that more than 60% of multi-exon human genes [3-5] and at least 40% of *Drosophila *genes [6] can produce differently spliced mRNA transcripts. The extent of alternative splicing of transcripts has led to suggestions that its purpose is to expand functional complexity in the cell [7,8] and that alternative splicing may be one of the keys to understanding the discrepancy between the number of genes and functional complexity [9]. Alternative splicing events within protein coding regions can generate a range of protein isoforms with altered structure and biological function [10-12] and, therefore, alternative splicing has the potential to expand the cellular protein repertoire. However, there is still some controversy about the degree of impact that individual alternative splicing events can have *in vivo *on the range of conventional protein functions [11,13].

Considerable supporting evidence exists for the expression of multiple alternative mRNA transcripts. The expression of many differently spliced mRNA transcripts is strongly supported by both microarray data [4] and by cDNA and expressed sequence tag (EST) sequence evidence [3]. There is overwhelming evidence for the expression of transcripts even when these might encode protein sequences with unusual evolutionary or structural features, but it is much more difficult to demonstrate the existence of alternative variants at the protein level. To date, most evidence for the translation of alternative splice variants as stable proteins has come from individual experiments. One well known example is the *Dscam *gene, which codes for an axon guidance receptor involved in the formation of synaptic branching patterns in neural circuit development [14]. It has four sets of mutually exclusive alternative exons that code for three immunoglobulin-like domains and a trans-membrane domain that could theoretically generate 38,016 different protein isoforms. It has been shown that the expression of different *Dscam *isoforms affects the recognition of mechanosensory neurons [15] and two *Dscam *isoforms have been crystallized [16]. Another important example in *Drosophila *is the *Sex lethal *gene. Alternative splicing of this gene determines whether the fly will be male or female [17]. *Sex lethal *encodes an RNA-binding protein that forms part of a complex regulatory cascade [18]. The male-specific isoform of *Sex lethal *is an inactive truncated protein.

Incontrovertible evidence for the expression of alternative protein variants ought to be available from proteomics technologies, but until recently these methods have only been able to identify a fraction of the peptide ions present in protease (tryptic) digests. This has hindered the analysis of protein isoforms. However, one recent study was able to show that 16 pairs of alternative protein isoforms were expressed in humans [19] based on peptide data from the Peptide Atlas [20]. Two recent large scale proteomics studies have generated extensive, high quality peptide catalogs from the *Drosophila melangaster *proteome. The first was able to match peptides to almost 7,000 proteins (50% of the *Drosophila *genome), a level of coverage that has not been reached for any other complex eukaryote [21]. It was achieved using a novel iterative strategy that maximized sample diversity. The second study detected phosphorylated peptides representing 3,500 *Drosophila *proteins [22]. The two studies are complementary; only a fraction of the peptides detected were present in both studies. Both studies also used the same protein database to assign peptide sequences to the generated tandem mass spectra, facilitating the comparison of the two datasets.

The extent and coverage of these two sets of peptides has allowed us to perform the first large-scale analysis of alternative splicing at the protein level. This analysis demonstrates that the expression of protein isoforms is widespread and points the way towards further research in this area. The results presented here should prompt further studies to generate and analyze proteomic data sets in the search for protein isoforms expressed in different organisms.

## Results and discussion

Our analysis was based on the peptides detected in two proteomics studies [21,22]. The 'Brunner set' consisted of 32,729 non-overlapping peptides from the *D. melangaster *proteome [21]. The 'Bodenmiller set' contained 10,118 high-confidence phosphorylated peptides [22]. There were significant differences in the collection methods. In the Brunner analysis the protein samples came from experiments carried out under a wide range of distinct conditions and developmental stages of *Drosophila*, while the samples used in the Bodenmiller analysis were from a single *Drosophila *cell line grown under just five different conditions. In both studies the peptides were identified by searching against *in silico *trypsin digests of the FlyBase *D. melangaster *proteome [23].

### Identifying splice isoform unique peptides

In the first step of analysis we searched for peptides that unambiguously indicated the presence of two or more splice isoforms from the same gene from the more than 42,000 peptides identified in the two proteomics studies. Peptides were matched to the proteins in FlyBase (release 5.4). Since FlyBase was used to identify the peptides from both studies, the peptides could be mapped back directly to proteins in FlyBase using a simple Perl script. Each of the more than 42,000 peptides mapped to at least one gene in FlyBase.

FlyBase release 5.4 contains 15,181 genes, of which 14,141 are predicted to be protein coding. The release contains a total of 20,823 protein coding transcripts and 17,961 of the polypeptide gene products are unique. The 2,762 transcripts that are alternatively spliced in the 3' or 5' untranslatable regions were not considered in this study, since they produce identical translated products and cannot be distinguished with peptide data alone. A total of 2,406 protein-coding genes (17.01%) code for more than one gene product.

It is important to note that the peptides in the Brunner and Bodenmiller experiments were identified using FlyBase. This means that neither of the studies could characterize peptides that did not match FlyBase sequences. As a result, our analysis of splice isoforms had to be limited to the alternative splice isoforms annotated in FlyBase. Therefore, the highest possible detectable alternative splicing rate from the two experiments was 17.01%.

**Figure 1:**
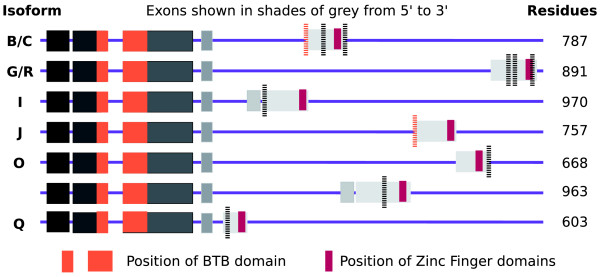
Schematic representation of the *LOLA *gene. The figure shows a representation of the seven variants detected by the two analyses. Coding exons are shown in shades of gray and the position of the BTB and zinc-finger domains are marked in color. Introns are not to scale. *LOLA *variants have an invariant amino-terminal region, but different carboxyl termini. Despite the differences, all these carboxy-terminal regions contain paired zinc-finger domains. Peptides detected for this gene are shown as vertical dashed lines, and are highlighted in orange when the peptide crosses the exon boundary. All the peptides detected in the two analyses were in the variable carboxy-terminal regions.

After matching the Brunner and Bodenmiller peptides to the unique proteins in FlyBase, we searched for genes that had two or more alternative isoforms confirmed by the peptide evidence. Genes with confirmed alternative protein isoforms were those for which it was possible to map peptides to regions unique to two or more alternative isoforms. In other words, where the detected peptides could unequivocally demonstrate the presence of two gene products with distinct protein sequences (for example, Figure 1).

The peptide evidence from the Brunner set confirmed multiple alternative isoforms for 76 genes and the evidence from the Bodenmiller set confirmed multiple alternative isoforms for 60 genes. There was a certain amount of overlap between the two experiments - 19 genes had multiple isoforms confirmed by the peptides from both analyses. In addition, when the two sets of peptides were combined there was evidence for alternative gene products from another 13 genes. In total, we were able to demonstrate that 130 separate *Drosophila *genes expressed at least two alternative isoforms (Additional data file 1). While this is only a small proportion of the genes that are supposed to express alternative protein isoforms, the figure is considerably higher than any previous study we know.

**Figure 2:**
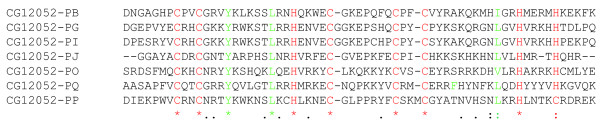
Alignment of *LOLA *isoforms. The alignment of the carboxy-terminal C_2_H_2 _DNA-binding zinc finger domains of the seven detected *LOLA *isoforms (CG12052). Six isoforms have two C_2_H_2 _zinc-finger domains, isoform PP has a C_2_H_2 _DNA-binding zinc-finger domain and a C_2_HC zinc finger domain. Zinc-binding residues (cysteines and histidines) are marked in red, and structurally important residues marked in green. The symbols below the alignment indicate the degree of conservation of the aligned residues: asterisk, completely conserved column; colon, highly conserved column; single dot, some conservation.

Those genes for which it was possible to show the presence of three or more distinct gene products were particularly interesting. Five genes - *SNF4A-gamma*, *Akap200*, *14-3-3-epsilon*, *mod(mdg4)*, and *LOLA *- each expressed at least four distinct gene products. By combining the peptide data it was possible to show that one gene, *LOLA*, expressed at least seven different isoforms (Figure 1). The 26 splice isoforms of *LOLA *annotated in Flybase have very different carboxyl termini, but two-thirds of them include two zinc-finger domains. All seven confirmed *LOLA *splice isoforms have both carboxy-terminal zinc-finger domains (Figure 2). Of the five genes shown to have more than two distinct gene products, there are individual studies for *LOLA *[24], *SNF4A-gamma *[25] and *mod(mdg4) *[26] that predict the presence of multiple splice isoforms.

There was no evidence of the expression of alternative splice isoforms of the *Dscam *protein that was mentioned in the introduction, but the two studies did show the expression of different isoforms of *Sex lethal *(for the tandem mass spectra of the phosphopeptides for the two isoforms see Additional data files 2-4). Other genes that are predicted to have differently functioning splice isoforms include *CTBP*, thought to be important in development and to have two variants that are conserved across all insect species [27], *Eif2 *and *Su(var)3-9*, which fuse in *Drosophila *and are expected to create two different gene products [28], and *Polychaetoid *[29] and *Thioredoxin reductase *[30], both of which are predicted to have gene products whose cellular locations are controlled by alternative splicing.

### Simulated peptide detection

While the two studies present irrefutable evidence that alternatively spliced transcripts are expressed as proteins, the total number of genes with confirmed alternative products from the two experiments is small. We could confirm the expression of alternative protein isoforms for just 1.1% of the 6,980 unambiguously identified genes in the Brunner set, while the Bodenmiller study showed that 1.8% of the 3,472 identified genes express more than one protein isoform.

However, this apparently low level of alternative splicing at the protein level has to be seen in the context of the relatively low coverage of the *Drosophila *proteome by the two experiments. Although the peptides detected by the Brunner study did confirm the presence of gene products for more than half of the *Drosophila *genes, the identified peptides covered just 5% of the amino acid residues in the *Drosophila *proteome. This obviously decreases the chances of finding peptides that unambiguously correspond to alternatively spliced regions.

In order to demonstrate whether the low rates of detection of alternative isoforms are significant, we carried out simulated *in silico *peptide identification experiments. These *in silico *experiments determine the expected rates of detection assuming that all peptides are equally detectable, even though some proteins may be more abundant or more easily detectable than others. The simulations cannot tell us what the real rate of alternative splicing at the protein level is, since the maximum detectable rate is limited by the rate of alternative splicing found in FlyBase (in this particular case 17%; see above). However, they do provide an estimation of the number of alternative isoforms that we would have expected the two experiments to detect.

For the comparison with the Brunner analysis we drew 37,279 peptides at random from an *in silico *trypsin digest of the *D. melangaster *proteome based on Flybase release 5.4. The simulation was performed 1,000 times. The results showed that a random selection of 37,279 peptides from the *in silico *digest would be expected to confirm the expression of alternatively spliced isoforms for a mean of 242.75 genes (standard deviation of 9.23). By way of contrast, the peptides identified in the Brunner analysis confirmed multiple alternatively spliced isoforms for just 76 genes.

For the Bodenmiller study we drew 10,118 peptides at random from the *in silico *trypsin digest and again the simulation was performed 1,000 times. From the random drawing we were able to show that 10,118 peptides would be expected to confirm expression of distinct splice isoforms for a mean of 56.24 genes (standard deviation of 5.05). The Bodenmiller analysis confirmed multiple alternatively spliced gene products for 60 genes.

The simulations allowed us to show that the Brunner analysis detects a little under a third of the number of genes that would be expected to produce alternative isoforms. There are several possible explanations for this. One reason may be that many isoforms are only expressed in certain tissues or certain stages of development. Given the wide range of cell types and developmental states that were used in this experiment, this explanation seems less likely. A second possibility is that the transcripts predicted from cDNA and EST evidence are simply not all transcribed and the transcript evidence is an overestimation of the real number of proteins expressed in the cell. Another explanation might be that many alternative isoforms may only be expressed in very low quantities and are less easily detected.

By way of contrast with the results from the Brunner simulations, the 60 genes with confirmed multiple alternatively spliced gene products in the Bodenmiller analysis is almost exactly what would be expected from the Bodenmiller simulations. It is somewhat surprising that it was the Bodenmiller analysis, and not the Brunner analysis, that detected the rate of alternative splicing expected from the random simulations. The prevailing ideology of alternative splicing assumes that alternative isoforms are expressed in distinct tissues and developmental stages; therefore, we would expect to confirm a higher rate of alternative splicing with the Brunner analysis, where many cell types and developmental stages were interrogated, than in the Bodenmiller analysis, where only a single cell type was tested.

The difference in the frequency of alternative splicing detected by the two studies is enlightening. The Bodenmiller analysis identified proportionally more alternative isoforms than the Brunner analysis (1.8% of genes detected in the Bodenmiller study had alternative protein isoforms, compared to 1.1% detected in the Brunner experiments) and this is even more clear from the simulations. The contrasting results from the two analyses strongly suggest the possibility that methods such as those used in the Bodenmiller analysis are more sensitive when it comes to detecting certain alternative isoforms.

**Figure 3:**
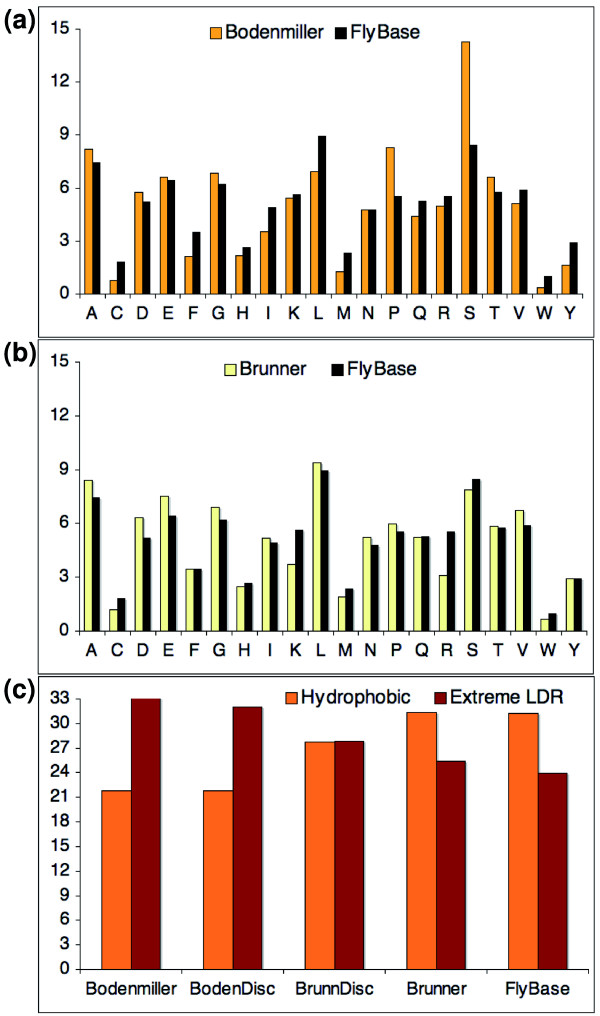
Composition of peptides identified in the Brunner and Bodenmiller studies. FlyBase residue composition was calculated from Flybase release 5.4. **(a) **Comparison of the percentage of each amino acid found in the Bodenmiller peptides and in the *Drosophila *proteome. **(b) **Comparison of the proportion of each amino acid in the Brunner peptides and the *Drosophila *proteome. The three sets of proteins differed most in the proportion of hydrophobic and disorder-promoting residues. **(c) **Comparison of the percentage of each type of residue in five different sets of peptides. Hydrophobic residues were C, F, I, L, M, V, W and Y. Disorder promoting residues (Extreme LDR) were A, D, E, G, P, N and S (according to Romero *et al*. [35]). BodenDisc is the subset of peptides that could be used to discriminate one isoform from another in the Bodenmiller analysis; BrunnDisc is the subset of peptides that could be used to discriminate one isoform from another in the Brunner analysis. The Brunner discriminating peptides had markedly fewer hydrophobic residues and markedly more disorder promoting residues than the whole set of Brunner peptides and the *Drosophila *proteome.

While some peptides were identified by both studies, on the whole the peptides recognized by the two analyses are different. This is clear from the residue composition of the peptides detected by the two analyses. Although the residue composition of the peptides found in the Brunner analysis is similar to the residue composition of the *Drosophila *proteome - except that there are fewer basic residues and more acidic residues (Figure 3b) - the peptides detected in the Bodenmiller analysis have a quite distinctive composition (Figure 3a). The Bodenmiller analysis specifically selected peptides that were phosphorylated. The peptides detected in these experiments have substantially more serine residues (unsurprising because almost 90% of the phosphorylated residues are serines), but also many more proline (5.5-7.6%), asparagine (4.7-5.7%), glycine (6.2-7.2%) and aspartate residues (5.2-5.9%). All these values are significantly higher than expected according to Chi-squared tests (*p*-values < 0.005).

In addition, all hydrophobic residues are markedly under-represented: the values for cysteine (1.83% of the residues in the *Drosophila *proteome and just 0.63% of the Bodenmiller peptides), phenylalanine (3.47 and 2.18%), isoleucine (4.9 and 3.71%), leucine (8.94 and 6.69%), methionine (2.33 and 1.14%), valine (5.9 and 5.34%), tryptophan (0.98 and 0.35%) and tyrosine (2.92 and 1.57%) are significantly less than expected (all Chi-squared *p*-values < 0.005). The peptides from the Bodenmiller set are considerably less hydrophobic than normal - just one in five of the Bodenmiller residues are hydrophobic, compared to the one in three residues in the *Drosophila *proteome (Figure 3c).

**Figure 4:**
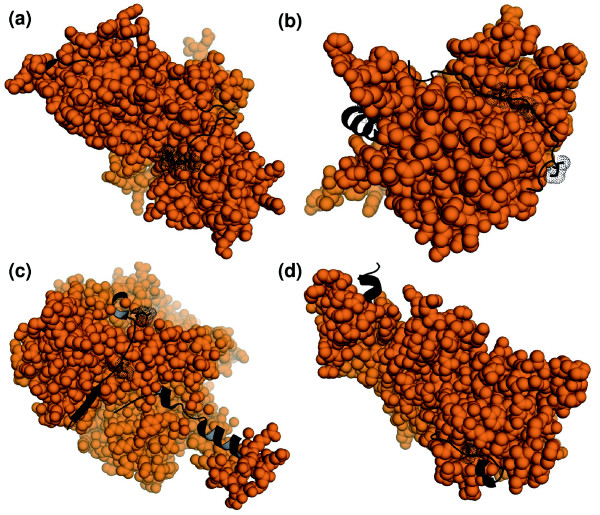
Mapping of detected phosphorylation sites to known structures. Peptides detected in the Bodenmiller analysis were mapped to highly similar, known structures. The three-dimensional structures are shown in orange spacefilling representation except where the peptides map to the structures (shown as black ribbons). Detected phosphorylation sites are shown as black dots. **(a) **The alternative isoforms generated from *shaggy *are 76% sequence identical to human glycogen synthase kinase 3 beta [PDB:1j1c]. The peptides detected in the analysis covered two regions. The amino-terminal region includes 22 residues that are known to be disordered in solution and are therefore not shown. **(b) **The structure for *Drosophila *fructose-1-biphosphate aldolase has been solved and the Bodenmiller analysis finds peptides covering two regions - both are on the surface of the structure. **(c) ***Drosophila *moesin has 75% identity to fall army worm moesin, for which the three-dimensional structure has been solved [PDB:2i1j]; in addition to the residues marked as found in the Bodenmiller analysis, a further 15 residues that were detected are not shown in the figure because they are disordered in solution. **(d) **The isoforms generated from *alphabet *are 52% identical to human phosphatase 2C [PDB:1a6q]. The analysis detected two peptides, one of which also coincided with the 14 disordered carboxy-terminal residues of the template (not shown).

This residue composition of the Bodenmiller peptides is typical of regions that are disordered in solution. It is well known that proteins with few hydrophobic residues and more polar residues are likely to correspond to disordered regions of the structure [31,32]. Studies also suggest that phosphorylated residues tend to be more frequent in flexible, unstructured segments and linkers [33,34]. Taken together, this information strongly suggests that many of the Bodenmiller peptides, as well as being in exposed regions on the surface of proteins, will be disordered when in solution. Indeed, where the Bodenmiller peptides can be mapped to known structures, most map to regions on the surface or regions known to be disordered in solution (Figure 4).

### False positive rates

It is widely recognized that a certain proportion of peptides identified in proteomics techniques can be false positives. However, both the Brunner and Bodenmiller studies have low rates of false positives. The Brunner analysis has a false positive rate of approximately 5% and the Bodenmiller analysis has a false positive rate of 1-4%. Even if 10% of these peptides were to be false positives (twice the determined value), there would still be considerably more than 100 genes with evidence of alternative splicing at the protein level from the two studies. In any case, a small number of false positives will not affect the main conclusions of this study. Most alternative transcripts do seem to produce alternative gene products and many of these alternative isoforms may have regions that are disordered in solution.

### Re-analysis of the spectra

Given the depth and range of peptides detected in the two studies, we might also have expected to be able to uncover the expression of peptides not in FlyBase, such as those from predicted genes and transcripts (isoforms), translated pseudogenes and small RNAs, or in principal any other peptide produced by the 6-frame translation of the fly genome. A complete re-analysis of the spectra from the Bodenmiller study was beyond the scope of this paper, but we were able to carry out an initial re-analysis against a locally generated database that contained 903,842 peptides from translated transcripts from predicted gene models, translated pseudogenes and translated miscellaneous functional RNA. The re-analysis identified seven peptides that mapped exclusively to predicted gene models and two peptides that were linked to the miscellaneous RNA. There was no evidence for the expression of any of the pseudogenes as peptides.

Three of the predicted gene models (genscan_masked:gene254366, genscan_masked:gene247065, and genscan_masked:gene245985) were not similar to any sequences in the UniProt database. One *ab initio *predicted gene model (genscan_masked:gene264127) did match a unique sequence in UniProt, but only because the prediction itself had been erroneously included in the UniProt sequence database.

One peptide mapped to four different predictions (genscan_masked:gene266459, genie_masked:gene1703010, genie_masked:gene1402427 and genscan_masked:gene1391762) that were 40% identical to a putative gag-pol protein (*Drosophila ananassae*). The remaining two predicted gene models identified by the spectra might be alternative variants of *vav *(both genie_masked:gene1736185 and genscan_masked:gene267148) and *lethal (2) 05510 *(genscan_masked:gene263593) but have yet to be annotated as variants in FlyBase.

Of the two miscellaneous transcripts identified by the re-analysis of the spectra, one is a piece of rRNA (FBtr0114214) that is not similar to anything in the UniProt database and the other maps to a piece of small nucleolar RNA (snoRNA; *Or-aca1*, FBtr0113530) that, when translated, is 71% identical (but over just 20 residues) to the hypothetical protein SNOG_09564 (*Phaeosphaeria nodorum SN15*). *Or-aca1 *is located inside an intron of *ribosomal protein S16*.

## Conclusion

### Genome-wide expression of alternative isoforms

We have been able to demonstrate conclusive evidence for the genome-wide expression of alternative splice variants at the protein level and have shown that distinct proteins are indeed produced from alternative splice variants. The results from the two large-scale proteomics studies on which our analysis is based showed that the expression of alternative gene products is extensive. These studies confirmed the presence of multiple alternative isoforms for over a hundred genes. Moreover, the alternative isoforms detected in these two studies were sufficiently stable *in vivo *and produced in sufficient quantities to be detectable in proteomics workflows. Even though the current technical limitations of proteomics studies allowed the recovery of just a small fraction of the potential alternative isoforms (less than 2% of *Drosophila *genes were identified with alternative protein isoforms), the results were enough to estimate the presence of alternative splicing in the genome and to propose that most, if not all, recorded alternative variants are likely to be expressed at the protein level in some form.

### Phosphopeptide detection techniques are more sensitive

The comparison of the two proteomics studies showed that the level of expression of alternatively spliced variants in the general proteomics analysis of Brunner was less than would be expected, but that the expression levels of alternative gene products in the Bodenmiller experiment, which specifically targeted and identified phosphopeptides, corresponded to the levels of expression predicted by FlyBase. The higher proportion of alternatively spliced gene products detected in the Bodenmiller analysis is most probably related to the sensitivity of the analysis of charged phosphopeptides. One of the effects of the sensitivity to phosphorylation sites is that the identified peptides have a significant compositional bias. The peptides have many fewer hydrophobic residues and markedly more polar residues, suggesting that many of these phosphorylation sites are in regions that are disordered in corresponding protein structures.

This observation is interesting since it may have consequences for our understanding of the structural and functional consequences of splicing. The detailed analysis of the potential effects of alternative splicing in proteins shows that alternative splicing would be expected to lead to substantial rearrangements in the corresponding structures [11] and it is unlikely that the large changes introduced by alternative splicing events will generate regions that fold with a stable hydrophobic core. It has previously been suggested that a substantial proportion of alternative gene products are unstructured in solution [35]. A corollary to this is that there are only eight known pairs of alternative splice isoforms in the Protein Data Bank (PDB) structural database [36]. In five of these pairs the regions resulting from alternative splicing events are disordered. It may be that many alternative splicing events result in proteins that are, at least in part, unstructured and flexible in solution. If alternative splicing events are related to disordered regions and phosphorylated residues are more frequent in these unstructured and flexible regions, then it follows that the disordered regions resulting from alternative splicing events will be more easily detected by methods that detect phosphorylated peptides. Therefore, it is not surprising that the Bodenmiller analysis was able to detect a higher proportion of splice isoforms.

The results of this analysis have shown that proteomics data can indeed be used to investigate the extent of alternative splicing at the protein level. The Bodenmiller analysis detected peptides that differed from those in the Brunner analysis because they specifically isolated phosphorylated peptides from whole cell lysate, suggesting a methodology for carrying out further experiments to detect alternative splicing at the protein level.

Perhaps surprisingly though, our initial re-analysis of the liquid chromatography tandem mass spectrometry data using databases of predicted transcripts, translated pseudogenes and small RNAs failed to reveal any significant new findings. Unfortunately, while we were able to detect some evidence for the expression of small RNAs and predicted gene models, the number of novel identified peptides fell within the estimated false positive rate. A complete re-analysis of the spectra might have produced more interesting results. However, our initial re-analysis made it clear that any proteomics study of the expression of functional aspects of the genome would be complicated by a series of logistical challenges. For example, six-frame translations of genome transcripts would create an enormous search space that would result in extensive and impracticable database search times. In addition, peptide detection sensitivity correlates with database size (especially if most added sequences are likely not to be real peptides) and is strongly reduced in the case of a 6-frame translated database.

Nevertheless, what is and is not expressed as protein is an interesting scientific question that needs to be addressed and our study points the way to further experiments of this nature. Future proteomics studies could address the question by searching against additional databases organized for the purpose, but specific methods to deal with the false positive problem still need to be developed and, ideally, the detected peptides would need to be confirmed using independent methods.

The fact that we can show that alternative transcripts are translated into proteins at the predicted rate is a great step forward and shows the importance of proteomics in validating predicted transcripts. Of course, showing that the alternative splice isoforms are indeed expressed as stable proteins is only the first step in assessing the functional role of alternative variants. In order to broaden our understanding of the role of genomic protein diversity, further experimental approaches are needed. We feel that these results will serve as an important point of reference for these experiments.

## Materials and methods

Our analysis was based on the peptides detected in two proteomics studies [21,22]. The first, the 'Brunner set' (for more details, see [21]) consisted of 32,729 non-overlapping peptides that could be uniquely attributed to a single gene product from the *D. melangaster *proteome. In total, the experimentally observed peptides contained sufficient information to identify 6,980 proteins unambiguously. The second dataset, the 'Bodenmiller set' (see [22] for more details) contained 10,118 high-confidence phosphorylated peptides from 3,472 gene models.

Even though the peptides detected in the two studies were identified based on tandem mass spectrometry, there were significant differences in the collection methods. In the Brunner analysis the protein samples from which the peptides were produced came from experiments carried out under a wide range of distinct conditions, including 5 developmental stages, 12 tissue types and 10 different fractionation techniques. Furthermore, the coverage was further augmented by a novel iterative data collection method [21] that resulted in a significant increase in coverage relative to previous studies. Cells used in the Bodenmiller analysis were from a single *Drosophila *cell line (Kc167), but cells were grown under five different conditions in order to maximize phosphorylation site identifications. Finally, phosphopeptides were isolated using three different phosphopeptide isolation methods [37] prior to mass spectrometric analysis using a high mass accuracy Fourier transform ion cyclotron resonance mass spectrometer in order to maximize the number of identified phosphopeptides.

In both studies the peptides were identified by searching against *in silico *trypsin digests of the FlyBase *D. melangaster *proteome. For both methods the false positive rate of peptide identification was assessed using the statistical tool Peptide Prophet [38] as well as a decoy database strategy and was found to be in the low percentage range. Most tandem mass spectra as well as their statistical analysis can be viewed in the PhosphoPep database [39].

In order to make estimates for the expected rate of alternative splicing, we carried out simulated peptide detection. For this *in silico *peptide detection experiment the *D. melangaster *proteome (dmel-all-translation-r5.4.fasta) was subject to an *in silico *trypsin digest. Peptides in the *in silico *digest were generated by cutting after arginine and lysine residues, except where they were followed by proline residues. We carried out 1,000 peptide detection simulations by drawing 10,118 peptides (for the Bodenmiller analysis) or 37,279 peptides (for the Brunner set) at random from the peptides generated *in silico*.

### Re-analysis of the spectra

We searched 154,509 spectra from the Bodenmiller dataset against a database that contained 903,842 peptides derived from 17,868 translated transcripts: 10, 000 translated transcripts came from predicted gene models from FlyBase, 1,456 came from the 6-frame translation of pseudogenes from FlyBase, 3,818 from 6-frame translations of miscellaneous functional RNA (rRNA, small nuclear RNA (snRNA) and snoRNA) from FlyBase and 2,594 were generated from the 6-frame translation of transcripts predicted by Manak *et al*. [40] to be functional.

## Abbreviations

EST: expressed sequence tag; PDB: Protein Data Bank; snoRNA: small nucleolar RNA.

## Authors' contributions

MLT designed and carried out the *in silico *experiments, analyzed the data and drafted the manuscript. BB performed the proteomics data analyses and contributed to the manuscript. RA designed the data collection and edited the manuscript. AV conceived of the study and edited the manuscript.

## Additional data files

The following additional data are available with the online version of this paper. Additional data file 1 lists genes with multiple isoforms detected in the Brunner and Bodenmiller studies. Additional data file 2 provides example tandem mass spectra of phosphopeptides distinguishing between the *Sex lethal *isoforms. Additional file 3 is a table listing detected ion masses for *Sex lethal *isoforms PD, PI and PL. Additional data file 4 is a table listing detected ion masses for *Sex lethal *isoforms PC, PG, PH, PJ, PN and PO.

## Supplementary Material

Additional data file 1 A list of all alternative isoforms confirmed by the Brunner and Bodenmiller analyses. Click here for file

Additional data file 2 Part 1A shows the phosphopeptide GFGMSHS*LPSGMSR, which is unique to the *Sex lethal *isoforms CG18350-PD, CG18350-PL, CG18350-PI. Part 1B shows the phosphopeptide GFGMS*HSLPSGMDTEFSFPSSSSR, which is unique to the *Sex lethal *isoforms CG18350-PG, CG18350-PH, CG18350-PO, CG18350-PC, CG18350-PJ, CG18350-PN. P is the Peptide Prophet score and corresponds to a <1% false positive rate. In addition, all fragment ion masses are shown and detected ions are highlighted in red. Click here for file

Additional data file 3 Fragment ion masses for the phosphopeptide GFGMSHS*LPSGMSR, which is unique to the *Sex lethal *isoforms CG18350-PD, CG18350-PL, CG18350-PI, are shown in tabular form. Detected ions are highlighted in red. Click here for file

Additional data file 4 Fragment ion masses for the phosphopeptide GFGMS*HSLPSGMDTEFSFPSSSSR, which is unique to the *Sex lethal *isoforms CG18350-PG, CG18350-PH, CG18350-PO, CG18350-PC, CG18350-PJ, CG18350-PN, are shown in tabular form. Detected ions are highlighted in red. Click here for file
