# Supplementary material for: Proteomics studies confirm the presence of alternative protein isoforms on a large scale
Source: Genome Biol. 2008 Nov 18;9(11):R162. doi: 10.1186/gb-2008-9-11-r162 (PMC2614494; doi:10.1186/gb-2008-9-11-r162)
Supplement: Additional data file 1 — A list of all alternative isoforms confirmed by the Brunner and Bodenmiller analyses. [file gb-2008-9-11-r162-S1.pdf]

Accession	Gene	GENE_ID	Total	Has Peptide	Isoforms Identified	Analysis
FBgn0000721	foraging	CG10033	2	4 of 10	CG10033-PI, -PA, -PB, -PH	Bodenmiller
FBgn0000721	foraging	CG10033	2	6 of 10	CG10033-PF, -PC, -PJ, -PD, -PE, -PG	Bodenmiller
FBgn0015602	BEAF-32	CG10159	2	1 of 2	CG10159-PA	Brunner
FBgn0015602	BEAF-32	CG10159	2	1 of 2	CG10159-PB	Brunner
FBgn0010415	Syndecan	CG10497	2	1 of 5	CG10497-PA	Brunner
FBgn0010415	Syndecan	CG10497	2	2 of 6	CG10497-PB, CG10497-PC	Brunner
FBgn0011661	Moesin	CG10701	2	4 of 10	CG10701-PJ, -PA, -PB, -PC	Bodenmiller
FBgn0011661	Moesin	CG10701	2	6 of 10	CG10701-PI, -PF, -PE, -PH, -PD, -PG	Bodenmiller
FBgn0034420	CG10737	CG10737	2	2 of 5	CG10737-PD, CG10737-PC	Bodenmiller
FBgn0034420	CG10737	CG10737	2	2 of 5	CG10737-PB, CG10737-PE	Brunner
FBgn0023388	Dap160	CG1099	2	1 of 2	CG1099-PA	Bodenmiller
FBgn0023388	Dap160	CG1099	2	1 of 2	CG1099-PB	Brunner
FBgn0031737	obstructor-E	CG11142	2	1 of 2	CG11142-PA	Brunner
FBgn0031737	obstructor-E	CG11142	2	1 of 2	CG11142-PB	Brunner
FBgn0034923	Upf3	CG11184	2	1 of 2	CG11184-PB	Bodenmiller
FBgn0034923	Upf3	CG11184	2	1 of 2	CG11184-PC	Bodenmiller
FBgn0035397	CG11486	CG11486	2	4 of 13	CG11486-PL, -PB, -PD, -PA	Bodenmiller
FBgn0035397	CG11486	CG11486	2	9 of 13	CG11486-PF, PH, PJ, PN, PG, PK, PE, PM, P	Bodenmiller
FBgn0015278	Pi3K68D	CG11621	2	1 of 3	CG11621-PB	Brunner
FBgn0015278	Pi3K68D	CG11621	2	2 of 3	CG11621-PC CG11621-PA	Brunner
FBgn0010352	Nc73EF	CG11661	2	2 of 8	CG11661-PH CG11661-PF	Brunner
FBgn0010352	Nc73EF	CG11661	2	6 of 8	CG11661-PG, -PC, -PB, -PA, -PI	Brunner
FBgn0037804	CG11870	CG11870	2	1 of 4	CG11870-PD	Bodenmiller
FBgn0037804	CG11870	CG11870	2	3 of 4	CG11870-PA CG11870-PC CG11870-PB	Brunner
FBgn0039635	CG11876	CG11876	2	2 of 4	CG11876-PA CG11876-PD	Brunner
FBgn0039635	CG11876	CG11876	2	2 of 4	CG11876-PC CG11876-PB	Brunner
FBgn0004167	karst	CG12008	2	1 of 3	CG12008-PA	Bodenmiller
FBgn0004167	karst	CG12008	2	1 of 3	CG12008-PC	Bodenmiller
FBgn0005630	longitudinals lacking	CG12052	7	1 of 26	CG12052-PI	Brunner
FBgn0005630	longitudinals lacking	CG12052	7	1 of 26	CG12052-PJ	Bodenmiller
FBgn0005630	longitudinals lacking	CG12052	7	1 of 26	CG12052-PO	Bodenmiller
FBgn0005630	longitudinals lacking	CG12052	7	1 of 26	CG12052-PP	Brunner
FBgn0005630	longitudinals lacking	CG12052	7	1 of 26	CG12052-PQ	Bodenmiller
FBgn0005630	longitudinals lacking	CG12052	7	2 of 26	CG12052-PC CG12052-PB	Brunner
FBgn0005630	longitudinals lacking	CG12052	7	2 of 26	CG12052-PG CG12052-PR	Brunner
FBgn0030052	CG12065	CG12065	2	2 of 4	CG12065-PA, CG12065-PB	Bodenmiller
FBgn0030052	CG12065	CG12065	2	2 of 4	CG12065-PC, CG12065-PD	Bodenmiller
FBgn0037303	CG12163	CG12163	2	1 of 2	CG12163-PA	Brunner
FBgn0037303	CG12163	CG12163	2	1 of 2	CG12163-PB	Brunner
FBgn0003660	Synaptobrevin	CG12210	2	1 of 2	CG12210-PA	Bodenmiller
FBgn0003660	Synaptobrevin	CG12210	2	1 of 2	CG12210-PB	Bodenmiller
FBgn0036515	CG12304	CG12304	2	1 of 2	CG12304-PA	Brunner
FBgn0036515	CG12304	CG12304	2	1 of 2	CG12304-PB	Brunner
FBgn0033668	CG13188	CG13188	2	1 of 2	CG13188-PA	Bodenmiller
FBgn0033668	CG13188	CG13188	2	1 of 2	CG13188-PB	Bodenmiller
FBgn0032036	CG13384	CG13384	2	1 of 6	CG13384-PC	Brunner
FBgn0032036	CG13384	CG13384	2	3 of 6	CG13384-PD, CG13384-PE CG13384-PF	Brunner
FBgn0027932	Akap200	CG13388	3	1 of 4	CG13388-PA	Bodenmiller/Brunner
FBgn0027932	Akap200	CG13388	3	1 of 4	CG13388-PD	Bodenmiller/Brunner
FBgn0027932	Akap200	CG13388	3	2 of 4	CG13388-PB, CG13388-PC	Bodenmiller/Brunner
FBgn0015907	bancal	CG13425	2	2 of 4	CG13425-PC, CG13425-PA	Bodenmiller
FBgn0015907	bancal	CG13425	2	1 of 4	CG13425-PD	Brunner
FBgn0031037	CG14207	CG14207	2	1 of 2	CG14207-PA	Brunner
FBgn0031037	CG14207	CG14207	2	1 of 2	CG14207-PB	Brunner
FBgn0004652	fruitless	CG14307	2	1 of 13	CG14307-PD	Bodenmiller

FBgn0004652	fruitless	CG14307	2	2 of 13	CG14307-PB, CG14307-PF	Bodenmiller
FBgn0031187	CG14619	CG14619	2	1 of 4	CG14619-PC	Bodenmiller
FBgn0031187	CG14619	CG14619	2	3 of 4	CG14619-PE, CG14619-PD, CG14619-PA	Bodenmiller
FBgn0035497	CG14995	CG14995	2	1 of 4	CG14995-PB	Bodenmiller
FBgn0035497	CG14995	CG14995	2	1 of 4	CG14995-PC	Bodenmiller
FBgn0035500	CG14998	CG14998	2	1 of 5	CG14998-PD	Bodenmiller
FBgn0035500	CG14998	CG14998	2	1 of 5	CG14998-PE	Bodenmiller
FBgn0028968	γCop	CG1528	2	1 of 2	CG1528-PA	Bodenmiller
FBgn0028968	γCop	CG1528	2	1 of 2	CG1528-PB	Bodenmiller
FBgn0030245	CG1637	CG1637	3	1 of 3	CG1637-PA	Brunner
FBgn0030245	CG1637	CG1637	3	1 of 3	CG1637-PB	Brunner
FBgn0030245	CG1637	CG1637	3	1 of 3	CG1637-PC	Brunner
FBgn0035348	CG16758	CG16758	2	1 of4	CG16758-PB	Brunner
FBgn0035348	CG16758	CG16758	2	1 of4	CG16758-PD	Brunner
FBgn0028671	Vha100-1	CG1709	3	1 of 8	CG1709-PG	Brunner
FBgn0028671	Vha100-1	CG1709	3	2 of 8	CG1709-PB, CG1709-PD	Brunner
FBgn0028671	Vha100-1	CG1709	3	2 of 8	CG1709-PC, CG1709-PE	Brunner
FBgn0001624	dlg1	CG1725	2	1 of 9	CG1725-PA	Bodenmiller
FBgn0001624	dlg1	CG1725	2	1 of 9	CG1725-PF	Brunner
FBgn0025803	SNF4Aγ	CG17299	4	1 of 8	CG17299-PF	Bodenmiller/Brunner
FBgn0025803	SNF4Aγ	CG17299	4	1 of 8	CG17299-PG	Bodenmiller/Brunner
FBgn0025803	SNF4Aγ	CG17299	4	2 of 8	CG17299-PB, CG17299-PA	Bodenmiller
FBgn0025803	SNF4Aγ	CG17299	4	2 of 8	CG17299-PC, CG17299-PE	Bodenmiller
FBgn0004435	Gα49B	CG17759	2	2 of 8	CG17759-PD, CG17759-PF	Brunner
FBgn0004435	Gα49B	CG17759	2	6 of 8	CG17759-PA, -PG, -PC, -PE, -PH, -PB	Brunner
FBgn0004907	14-3-3Z	CG17870	2	3 of 10	CG17870-PD, CG17870-PE, CG17870-PJ	Bodenmiller/Brunner
FBgn0004907	14-3-3Z	CG17870	2	5 of 10	CG17870-PH, -PG, -PA, -PB, -PI	Bodenmiller/Brunner
FBgn0086783	Myosin heavy chain	CG17927	2	1 of 13	CG17927-PF	Brunner
FBgn0086783	Myosin heavy chain	CG17927	2	1 of 13	CG17927-PK	Brunner
FBgn0013733	short stop	CG18076	3	1 of 6	CG18076-PH	Bodenmiller
FBgn0013733	short stop	CG18076	3	2 of 6	CG18076-PB, CG18076-PG	Bodenmiller
FBgn0013733	short stop	CG18076	3	2 of 6	CG18076-PE, CG18076-PA	Bodenmiller
FBgn0024277	trio	CG18214	3	1 of 5	CG18214-PD	Brunner
FBgn0024277	trio	CG18214	3	2 of 5	CG18214-PB, CG18214-PF	Bodenmiller/Brunner
FBgn0024277	trio	CG18214	3	2 of 5	CG18214-PC, CG18214-PA	Bodenmiller/Brunner
FBgn0014133	bifocal	CG1822	2	1 of 2	CG1822-PB	Brunner
FBgn0014133	bifocal	CG1822	2	1 of 2	CG1822-PC	Brunner
FBgn0003659	Sex lethal	CG18350	2	3 of 9	CG18350-PD, CG18350-PL, CG18350-PI	Bodenmiller
FBgn0003659	Sex lethal	CG18350	2	6 of 9	CG18350-PG, -PH, -PO, -PC, -PJ, -PN	Bodenmiller
FBgn0033504	CAP	CG18408	2	1 of 10	CG18408-PA	Bodenmiller
FBgn0033504	CAP	CG18408	2	1 of 10	CG18408-PB	Bodenmiller
FBgn0003870	tramtrack	CG1856	2	3 of 6	CG1856-PE CG1856-PB CG1856-PA	Brunner
FBgn0003870	tramtrack	CG1856	2	3 of 6	CG1856-PF CG1856-PC CG1856-PD	Brunner
FBgn0039672	alphabet	CG1906	2	1 of 5	CG1906-PB	Bodenmiller
FBgn0039672	alphabet	CG1906	2	3 of 5	CG1906-PA, CG1906-PC, CG1906-PD	Bodenmiller
FBgn0020621	Pkn	CG2049	2	2 of 5	CG2049-PC, CG2049-PD	Bodenmiller
FBgn0020621	Pkn	CG2049	2	2 of 5	CG2049-PF, CG2049-PB	Bodenmiller
FBgn0020653	Trxr-1	CG2151	2	1 of 2	CG2151-PB	Bodenmiller
FBgn0020653	Trxr-1	CG2151	2	1 of 2	CG2151-PC	Bodenmiller
FBgn0027280	lethal (1) G0193	CG2206	2	1 of 2	CG2206-PA	Brunner
FBgn0027280	lethal (1) G0193	CG2206	2	1 of 2	CG2206-PB	Brunner
FBgn0027951	MTA1-like	CG2244	2	1 of 2	CG2244-PB	Bodenmiller
FBgn0027951	MTA1-like	CG2244	2	1 of 2	CG2244-PA	Brunner
FBgn0033484	CG2269	CG2269	2	1 of 3	CG2269-PB	Brunner
FBgn0033484	CG2269	CG2269	2	2 of 3	CG2269-PC CG2269-PA	Brunner
FBgn0003371	shaggy	CG2621	2	1 of 11	CG2621-PD	Bodenmiller

FBgn0003371	shaggy	CG2621	2	8 of 11	CG2621-PB, -PH, -PC, -PF, -PI, -PE, -PJ, -PA	Bodenmiller
FBgn0001123	G-sα60A	CG2835	2	1 of 3	CG2835-PB	Brunner
FBgn0001123	G-sα60A	CG2835	2	2 of 3	CG2835-PC CG2835-PA	Brunner
FBgn0025726	unc-13	CG2999	2	1 of 3	CG2999-PB	Bodenmiller
FBgn0025726	unc-13	CG2999	2	2 of 3	CG2999-PC, CG2999-PA	Bodenmiller
FBgn0050035	CG30035	CG30035	2	1 of 2	CG30035-PA	Brunner
FBgn0050035	CG30035	CG30035	2	1 of 2	CG30035-PB	Brunner
FBgn0050084	CG30084	CG30084	2	1 of 4	CG30084-PA	Bodenmiller/Brunner
FBgn0050084	CG30084	CG30084	2	1 of 4	CG30084-PF	Bodenmiller/Brunner
FBgn0003638	su(wa)	CG3019	2	1 of 3	CG3019-PA	Brunner
FBgn0003638	su(wa)	CG3019	2	2 of 3	CG3019-PB CG3019-PC	Brunner
FBgn0043792	CG30427	CG30427	3	1 of 4	CG30427-PB	Brunner
FBgn0043792	CG30427	CG30427	3	1 of 4	CG30427-PC	Brunner
FBgn0043792	CG30427	CG30427	3	2 of 4	CG30427-PA CG30427-PD	Brunner
FBgn0051064	CG31064	CG31064	2	1 of 3	CG31064-PB	Brunner
FBgn0051064	CG31064	CG31064	2	1 of 3	CG31064-PE	Brunner
FBgn0051120	CG31120	CG31120	2	1 of 2	CG31120-PA	Brunner
FBgn0051120	CG31120	CG31120	2	1 of 2	CG31120-PB	Brunner
FBgn0024242	dystrophin	CG31175	2	1 of 8	CG31175-PB	Brunner
FBgn0024242	dystrophin	CG31175	2	4 of 8	CG31175-PF, -PG, -PH, -PA	Brunner
FBgn0020238	14-3-3?	CG31196	4	1 of 4	CG31196-PA	Bodenmiller/Brunner
FBgn0020238	14-3-3?	CG31196	4	1 of 4	CG31196-PB	Bodenmiller/Brunner
FBgn0020238	14-3-3?	CG31196	4	1 of 4	CG31196-PC	Bodenmiller/Brunner
FBgn0020238	14-3-3?	CG31196	4	1 of 4	CG31196-PD	Bodenmiller/Brunner
FBgn0003177	polychaetoid	CG31349	3	1 of 5	CG31349-PA	Bodenmiller/Brunner
FBgn0003177	polychaetoid	CG31349	3	1 of 5	CG31349-PE	Bodenmiller/Brunner
FBgn0003177	polychaetoid	CG31349	3	2 of 5	CG31349-PF, CG31349-PB	Bodenmiller
FBgn0051363	Jupiter	CG31363	2	4 of 6	CG31363-PH,-PE,-PC,-PD	Bodenmiller
FBgn0051363	Jupiter	CG31363	2	1 of 6	CG31363-PB	Brunner
FBgn0005666	bent	CG32019	2	1 of 4	CG32019-PC	Brunner
FBgn0005666	bent	CG32019	2	1 of 4	CG32019-PD	Brunner
FBgn0036518	RhoGAP71E	CG32149	2	1 of 3	CG32149-PA	Bodenmiller
FBgn0036518	RhoGAP71E	CG32149	2	1 of 3	CG32149-PC	Brunner
FBgn0005536	Mbs	CG32156	2	1 of 4	CG32156-PE	Bodenmiller
FBgn0005536	Mbs	CG32156	2	3 of 4	CG32156-PB, CG32156-PA, CG32156-PC	Bodenmiller
FBgn0000541	Enhancer of bithorax	CG32346	2	1 of 3	CG32346-PC	Bodenmiller
FBgn0000541	Enhancer of bithorax	CG32346	2	2 of 3	CG32346-PB, CG32346-PA	Bodenmiller
FBgn0052423	alan shepard	CG32423	2	1 of 3	CG32423-PA	Bodenmiller
FBgn0052423	alan shepard	CG32423	2	2 of 3	CG32423-PB, CG32423-PD	Bodenmiller
FBgn0013576	l(3)82Fd	CG32464	4	1 of 13	CG32464-PF	Bodenmiller
FBgn0013576	l(3)82Fd	CG32464	4	1 of 13	CG32464-PG	Bodenmiller/Brunner
FBgn0013576	l(3)82Fd	CG32464	4	1 of 13	CG32464-PK	Brunner
FBgn0013576	l(3)82Fd	CG32464	4	4 of 13	CG32464-PJ, -PN, -PL, -PB	Bodenmiller
FBgn0002781	modifier of mdg4	CG32491	7	1 of 29	CG32491-PA	Bodenmiller
FBgn0002781	modifier of mdg4	CG32491	7	1 of 29	CG32491-PO	Bodenmiller
FBgn0002781	modifier of mdg4	CG32491	7	1 of 29	CG32491-PP	Brunner
FBgn0002781	modifier of mdg4	CG32491	7	1 of 29	CG32491-PS	Brunner
FBgn0002781	modifier of mdg4	CG32491	7	1 of 29	CG32491-PT	Bodenmiller/Brunner
FBgn0002781	modifier of mdg4	CG32491	7	1 of 29	CG32491-PU	Bodenmiller
FBgn0002781	modifier of mdg4	CG32491	7	1 of 29	CG32491-PV	Brunner
FBgn0052549	CG32549	CG32549	2	1 of 6	CG32549-PA	Brunner
FBgn0052549	CG32549	CG32549	2	1 of 6	CG32549-PE	Brunner
FBgn0027066	Eb1	CG3265	2	1 of 5	CG3265-PE	Bodenmiller
FBgn0027066	Eb1	CG3265	2	4 of 5	CG3265-PB, -PC, -PA, -PD	Bodenmiller
FBgn0053113	Rtnl1	CG33113	3	1 of 7	CG33113-PA	Bodenmiller/Brunner
FBgn0053113	Rtnl1	CG33113	3	1 of 7	CG33113-PE CG33113-PB	Brunner

FBgn0053113	Rtnl1	CG33113	3	1 of 7		Bodenmiller/Brunner
FBgn0022085	lethal (2) k07433	CG33130	2	1 of 3	CG33130-PC	Bodenmiller
FBgn0022085	lethal (2) k07433	CG33130	2	2 of 3	CG33130-PB, CG33130-PA	Bodenmiller
FBgn0013263	Trithorax-like	CG33261	2	6 of 9	CG33261-PE, -PC, -PB, -PH, -PG, -PD	Bodenmiller
FBgn0013263	Trithorax-like	CG33261	2	3 of 9	CG33261-PA, CG33261-PF, CG33261-PI	Brunner
FBgn0053523	CG33523	CG33523	2	1 of 4	CG33523-PC	Brunner
FBgn0053523	CG33523	CG33523	2	1 of 4	CG33523-PD	Brunner
FBgn0053553	Darkener of apricot	CG33553	2	1 of 3	CG33553-PE	Bodenmiller/Brunner
FBgn0053553	Darkener of apricot	CG33553	2	2 of 3	CG33553-PA, CG33553-PB	Bodenmiller/Brunner
FBgn0053555	Bitesize	CG33555	2	1 of 7	CG33555-PC	Brunner
FBgn0053555	Bitesize	CG33555	2	2 of 7	CG33555-PA, CG33555-PB	Brunner
FBgn0053715	Msp-300	CG33715	2	1 of 3	CG33715-PD	Bodenmiller/Brunner
FBgn0053715	Msp-300	CG33715	2	1 of 3	CG33715-PE	Bodenmiller/Brunner
FBgn0000256	cappuccino	CG3399	2	1 of 9	CG3399-PA	Brunner
FBgn0000256	cappuccino	CG3399	2	1 of 9	CG3399-PD	Brunner
FBgn0026875	CG3638	CG3638	2	2 of 4	CG3638-PB, CG3638-PA	Bodenmiller
FBgn0026875	CG3638	CG3638	2	2 of 4	CG3638-PC, CG3638-PD	Bodenmiller
FBgn0004551	Ca-P60A	CG3725	2	1 of 8	CG3725-PA	Brunner
FBgn0004551	Ca-P60A	CG3725	2	1 of 8	CG3725-PH	Brunner
FBgn0022343	CG3760	CG3760	2	1 of 2	CG3760-PA	Bodenmiller/Brunner
FBgn0022343	CG3760	CG3760	2	1 of 2	CG3760-PB	Bodenmiller/Brunner
FBgn0033786	CG3884	CG3884	2	1 of 2	CG3884-PA	Brunner
FBgn0033786	CG3884	CG3884	2	1 of 2	CG3884-PB	Brunner
FBgn0035059	CG3894	CG3894	2	1 of 2	CG3894-PB	Bodenmiller
FBgn0035059	CG3894	CG3894	2	1 of 2	CG3894-PA	Brunner
FBgn0014141	cheerio	CG3937	2	1 of 4	CG3937-PD	Bodenmiller
FBgn0014141	cheerio	CG3937	2	1 of 4	CG3937-PA	Brunner
FBgn0004227	nonA	CG4211	2	1 of 3	CG4211-PA	Brunner
FBgn0004227	nonA	CG4211	2	1 of 3	CG4211-PB	Brunner
FBgn0259111	Ndae1	CG42253	2	1 of 3	CG4675-PA	Brunner
FBgn0259111	Ndae1	CG42253	2	1 of 3	CG4675-PB	Brunner
FBgn0000667	α actinin	CG4376	3	1 of 3	CG4376-PA	Brunner
FBgn0000667	α actinin	CG4376	3	1 of 3	CG4376-PB	Brunner
FBgn0000667	α actinin	CG4376	3	1 of 3	CG4376-PC	Brunner
FBgn0010256	Rbp2	CG4429	2	1 of 3	CG4429-PC	Bodenmiller
FBgn0010256	Rbp2	CG4429	2	2 of 3	CG4429-PA, CG4429-PB	Bodenmiller
FBgn0013765	centrosomin	CG4832	3	1 of 5	CG4832-PC	Bodenmiller
FBgn0013765	centrosomin	CG4832	3	1 of 5	CG4832-PA	Brunner
FBgn0013765	centrosomin	CG4832	3	1 of 5	CG4832-PB	Bodenmiller
FBgn0003721	Tropomyosin 1	CG4898	4	1 of 12	CG4898-PF	Brunner
FBgn0003721	Tropomyosin 1	CG4898	4	1 of 12	CG4898-PH	Bodenmiller
FBgn0003721	Tropomyosin 1	CG4898	4	1 of 12	CG4898-PK	Bodenmiller/Brunner
FBgn0003721	Tropomyosin 1	CG4898	4	1 of 12	CG4898-PA	Bodenmiller
FBgn0002938	ninaC	CG5125	2	1 of 2	CG5125-PA	Brunner
FBgn0002938	ninaC	CG5125	2	1 of 2	CG5125-PB	Brunner
FBgn0034345	CG5174	CG5174	2	3 of 7	CG5174-PA, CG5174-PB, CG5174-PJ	Bodenmiller
FBgn0034345	CG5174	CG5174	2	3 of 7	CG5174-PH, CG5174-PG, CG5174-PI	Bodenmiller
FBgn0034886	Pde8	CG5411	3	1 of 5	CG5411-PB	Brunner
FBgn0034886	Pde8	CG5411	3	1 of 5	CG5411-PD	Bodenmiller
FBgn0034886	Pde8	CG5411	3	2 of 5	CG5411-PE CG5411-PA	Brunner
FBgn0026178	scribbled	CG5462	2	2 of 6	CG5462-PA, CG5462-PB	Bodenmiller
FBgn0026178	scribbled	CG5462	2	2 of 6	CG5462-PI, CG5462-PD	Brunner
FBgn0002174	l(2)tid	CG5504	2	1 of 3	CG5504-PB	Brunner
FBgn0002174	l(2)tid	CG5504	2	1 of 3	CG5504-PC	Brunner
FBgn0038684	CG5558	CG5558	2	1 of 2	CG5558-PA	Brunner
FBgn0038684	CG5558	CG5558	2	1 of 2	CG5558-PB	Brunner

FBgn0010575	scribbler	CG5580	2	1 of 3	CG5580-PA	Bodenmiller
FBgn0010575	scribbler	CG5580	2	1 of 3	CG5580-PB	Bodenmiller
FBgn0011225	jaguar	CG5695	2	1 of 6	CG5695-PC	Bodenmiller
FBgn0011225	jaguar	CG5695	2	1 of 6	CG5695-PD	Bodenmiller
FBgn0032455	CG5792	CG5792	2	1 of 2	CG5792-PA	Brunner
FBgn0032455	CG5792	CG5792	2	1 of 2	CG5792-PB	Brunner
FBgn0003149	Paramyosin	CG5939	2	2 of 4	CG5939-PB CG5939-PA	Brunner
FBgn0003149	Paramyosin	CG5939	2	2 of 4	CG5939-PC CG5939-PD	Brunner
FBgn0000064	Aldolase	CG6058	2	1 of 8	CG6058-PH	Bodenmiller
FBgn0000064	Aldolase	CG6058	2	4 of 8	CG6058-PC, -PD, -PB, -PF	Bodenmiller
FBgn0036182	CG6084	CG6084	2	1 of 2	CG6084-PA	Bodenmiller/Brunner
FBgn0036182	CG6084	CG6084	2	1 of 2	CG6084-PB	Bodenmiller/Brunner
FBgn0033853	CG6145	CG6145	2	1 of 3	CG6145-PC	Bodenmiller
FBgn0033853	CG6145	CG6145	2	1 of 3	CG6145-PA	Brunner
FBgn0003600	Su(var)3-9	CG6476	2	1 of 3	CG6476-PA	Bodenmiller
FBgn0003600	Su(var)3-9	CG6476	2	2 of 3	CG6476-PC, CG6476-PB	Bodenmiller
FBgn0020439	fau	CG6544	3	1 of 4	CG6544-PA	Brunner
FBgn0020439	fau	CG6544	3	1 of 4	CG6544-PB	Brunner
FBgn0020439	fau	CG6544	3	1 of 4	CG6544-PC	Brunner
FBgn0000451	ectodermal	CG6611	2	1 of 3	CG6611-PC	Brunner
FBgn0000451	ectodermal	CG6611	2	2 of 3	CG6611-PA CG6611-PB	Brunner
FBgn0000462	dorsal	CG6667	2	1 of 3	CG6667-PC	Brunner
FBgn0000462	dorsal	CG6667	2	2 of 3	CG6667-PA CG6667-PB	Brunner
FBgn0035906	CG6673	CG6673	2	1 of 2	CG6673-PA	Brunner
FBgn0035906	CG6673	CG6673	2	1 of 2	CG6673-PB	Brunner
FBgn0004028	wings up A	CG7178	4	1 of 7	CG7178-PA	Brunner
FBgn0004028	wings up A	CG7178	4	1 of 7	CG7178-PB	Brunner
FBgn0004028	wings up A	CG7178	4	1 of 7	CG7178-PG	Brunner
FBgn0004028	wings up A	CG7178	4	2 of 7	CG7178-PD CG7178-PE	Brunner
FBgn0037137	Nopp140	CG7421	2	1 of 2	CG7421-PA	Brunner
FBgn0037137	Nopp140	CG7421	2	1 of 2	CG7421-PB	Brunner
FBgn0030991	CG7453	CG7453	2	1 of 2	CG7453-PA	Brunner
FBgn0030991	CG7453	CG7453	2	1 of 2	CG7453-PB	Brunner
FBgn0010349	Dhc64C	CG7507	2	1 of 2	CG7507-PA	Brunner
FBgn0010349	Dhc64C	CG7507	2	1 of 2	CG7507-PB	Brunner
FBgn0020496	CtBP	CG7583	2	1 of 5	CG7583-PE	Bodenmiller
FBgn0020496	CtBP	CG7583	2	4 of 5	CG7583-PA, -PC, -PB, -PD	Bodenmiller
FBgn0042693	PP2A-B	CG7913	2	1 of 4	CG7913-PB	Bodenmiller
FBgn0042693	PP2A-B	CG7913	2	2 of 4	CG7913-PD, CG7913-PE	Bodenmiller
FBgn0035835	ect4	CG7915	2	1 of 2	CG7915-PA	Bodenmiller
FBgn0035835	ect4	CG7915	2	1 of 2	CG7915-PB	Bodenmiller
FBgn0026193	par-1	CG8201	3	3 of 15	CG8201-PB, CG8201-PA	Bodenmiller
FBgn0026193	par-1	CG8201	3	5 of 15	CG8201-PQ, -PH, -PI, -PJ, -PO	Bodenmiller
FBgn0026193	par-1	CG8201	3	6 of 15	CG8201-PE, -PM, -PF, -PD, -PG, -PC	Bodenmiller
FBgn0033339	sec31	CG8266	2	1 of 2	CG8266-PA	Bodenmiller/Brunner
FBgn0033339	sec31	CG8266	2	1 of 2	CG8266-PB	Bodenmiller/Brunner
FBgn0033714	gartenzwerg	CG8487	2	1 of 2	CG8487-PA	Bodenmiller
FBgn0033714	gartenzwerg	CG8487	2	1 of 2	CG8487-PB	Bodenmiller
FBgn0013334	Sap47	CG8884	2	1 of 8	CG8884-PF	Bodenmiller
FBgn0013334	Sap47	CG8884	2	6 of 8	CG8884-PH, -PC, -PE, -PA, -PD, -PB	Bodenmiller
FBgn0014391	stunted	CG9032	2	1 of 2	CG9032-PA	Brunner
FBgn0014391	stunted	CG9032	2	1 of 2	CG9032-PB	Brunner
FBgn0001128	Gpdh	CG9042	2	1 of 3	CG9042-PB	Brunner
FBgn0001128	Gpdh	CG9042	2	1 of 3	CG9042-PC	Brunner
FBgn0015777	nervana 2	CG9261	2	2 of 5	CG9261-PC CG9261-PF	Brunner
FBgn0015777	nervana 2	CG9261	2	3 of 5	CG9261-PD CG9261-PA CG9261-PE	Brunner

FBgn0004873	hu li tai shao	CG9325	3	1 of 6	CG9325-PA	Brunner
FBgn0004873	hu li tai shao	CG9325	3	1 of 6	CG9325-PC	Brunner
FBgn0004873	hu li tai shao	CG9325	3	3 of 6	CG9325-PB CG9325-PE CG9325-PF	Brunner
FBgn0010482	l(2)01289	CG9432	2	1 of 4	CG9432-PA	Brunner
FBgn0010482	l(2)01289	CG9432	2	2 of 4	CG9432-PB CG9432-PD	Brunner
FBgn0034603	Glycogenin	CG9480	2	1 of 2	CG9480-PA	Bodenmiller/Brunner
FBgn0034603	Glycogenin	CG9480	2	1 of 2	CG9480-PB	Bodenmiller/Brunner
FBgn0003089	pipe	CG9614	2	1 of 12	CG9614-PF	Brunner
FBgn0003089	pipe	CG9614	2	1 of 12	CG9614-PK	Brunner
FBgn0015600	toucan	CG9660	2	1 of 2	CG9660-PA	Bodenmiller/Brunner
FBgn0015600	toucan	CG9660	2	1 of 2	CG9660-PB	Bodenmiller/Brunner
FBgn0020306	domino	CG9696	2	1 of 3	CG9696-PE	Bodenmiller
FBgn0020306	domino	CG9696	2	2 of 3	CG9696-PA, CG9696-PD	Bodenmiller
FBgn0030749	Annexin B11	CG9968	2	1 of 2	CG9968-PA	Brunner
FBgn0030749	Annexin B11	CG9968	2	1 of 2	CG9968-PB	Brunner
