# Supplementary figures and images for: Proteomics studies confirm the presence of alternative protein isoforms on a large scale
Source: Genome Biol. 2008 Nov 18;9(11):R162. doi: 10.1186/gb-2008-9-11-r162 (PMC2614494; doi:10.1186/gb-2008-9-11-r162)

**A**

1e+04

Library

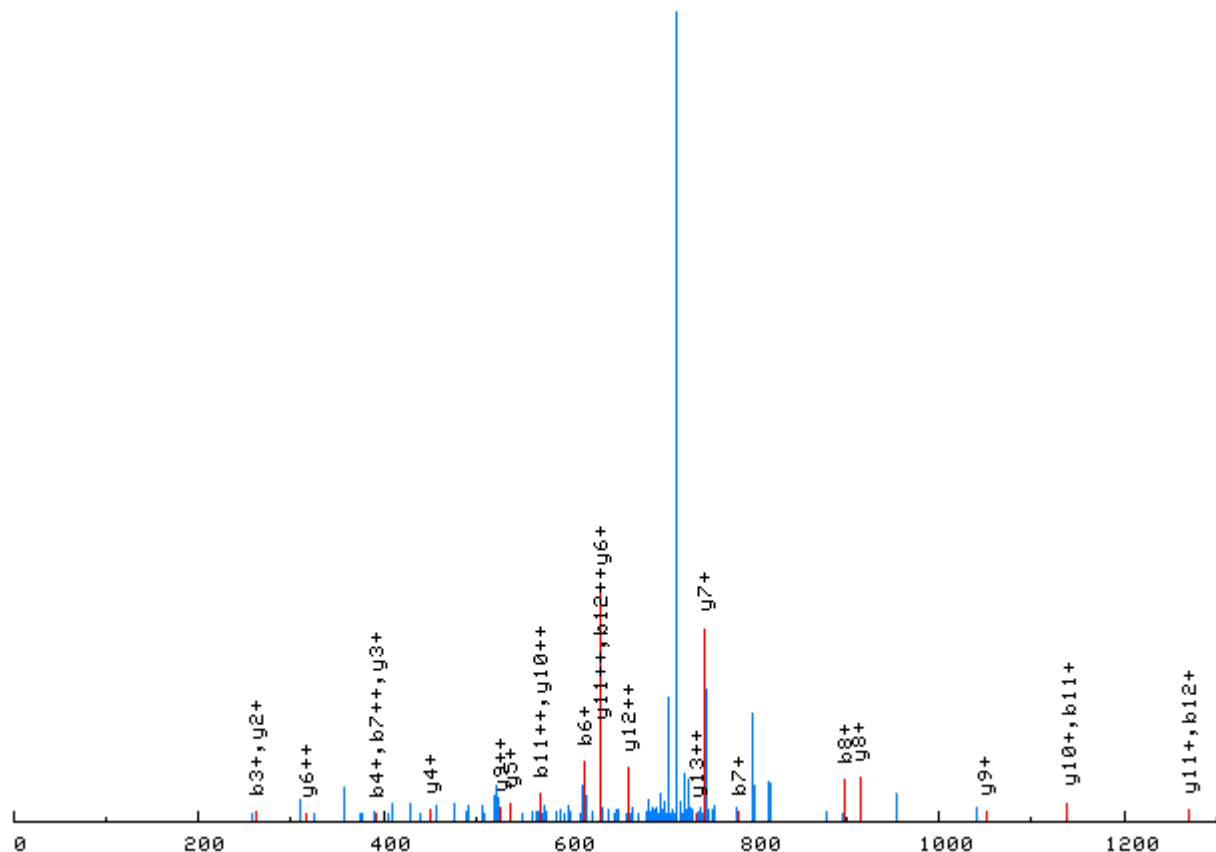

GFGMSHS<sup>167</sup>LP SGMSR/2 (P = 0.9900)

**B**

1e+04

Library

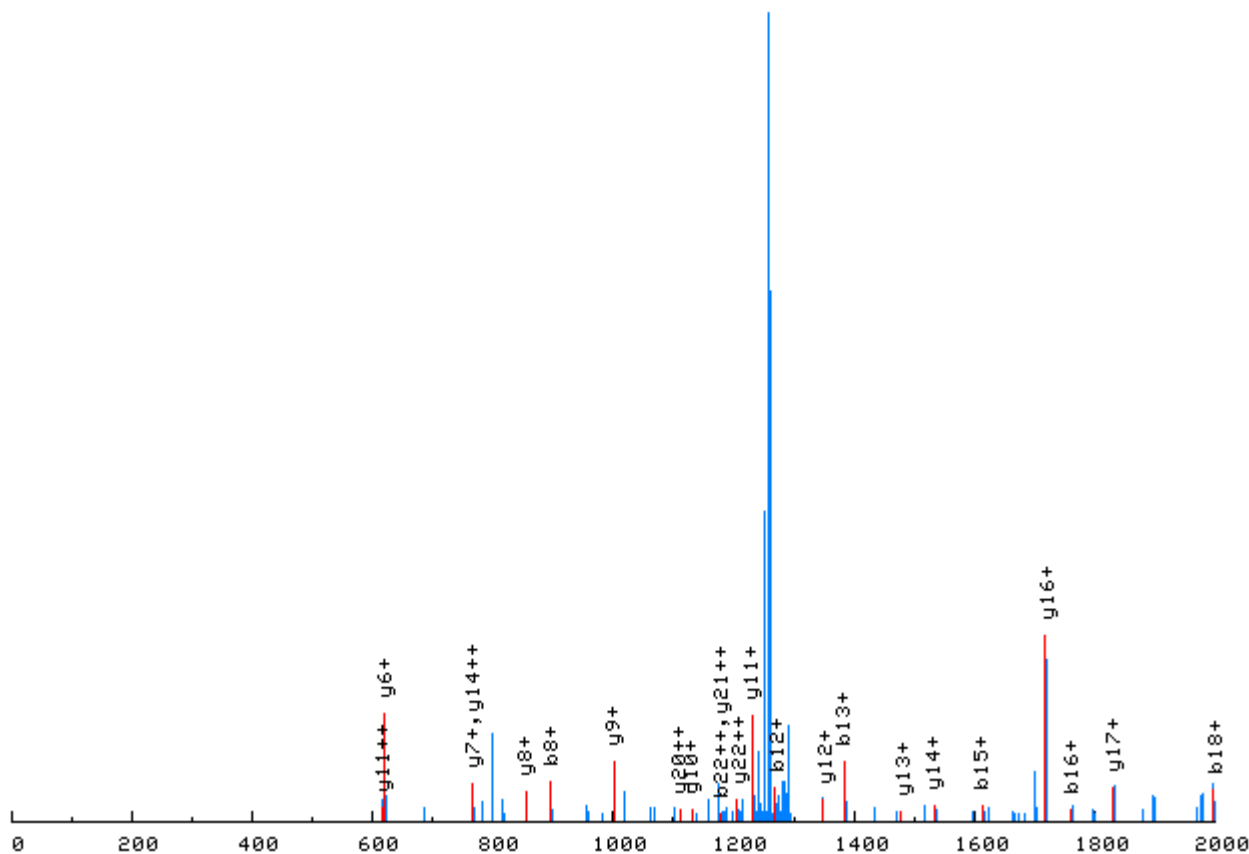

GFGMS<sup>167</sup>HSLPSGMDTEFSFPSSSR/2 (P = 0.9162)

Supplement: Additional data file 2 — Part 1A shows the phosphopeptide GFGMSHS*LPSGMSR, which is unique to the Sex lethal isoforms CG18350-PD, CG18350-PL, CG18350-PI. Part 1B shows the phosphopeptide GFGMS*HSLPSGMDTEFSFPSSSSR, which is unique to the Sex lethal isoforms CG18350-PG, CG18350-PH, CG18350-PO, CG18350-PC, CG18350-PJ, CG18350-PN. P is the Peptide Prophet score and corresponds to a <1% false positive rate. In addition, all fragment ion masses are shown and detected ions are highlighted in red. [file gb-2008-9-11-r162-S2.pdf]
