# Supplementary material for: Proteomics studies confirm the presence of alternative protein isoforms on a large scale
Source: Genome Biol. 2008 Nov 18;9(11):R162. doi: 10.1186/gb-2008-9-11-r162 (PMC2614494; doi:10.1186/gb-2008-9-11-r162)
Supplement: Additional data file 3 — Fragment ion masses for the phosphopeptide GFGMSHS*LPSGMSR, which is unique to the Sex lethal isoforms CG18350-PD, CG18350-PL, CG18350-PI, are shown in tabular form. Detected ions are highlighted in red. [file gb-2008-9-11-r162-S3.pdf]

b^{1+}	b^{2+}	#	AA	#	y^{1+}	y^{2+}
58.0593	29.5333	**1**	**G**	**14**		
205.2359	103.1216	**2**	**F**	**13**	1474.5856	**737.7965**
**262.2878**	131.6476	**3**	**G**	**12**	1327.4090	**664.2082**
**393.4804**	197.2439	**4**	**M**	**11**	**1270.3571**	**635.6823**
480.5586	240.7830	**5**	**S**	**10**	**1139.1645**	**570.0860**
**617.6997**	309.3535	**6**	**H**	**9**	**1052.0863**	**526.5469**
**784.7578**	**392.8826**	**7**	**S[167]**	**8**	**914.9452**	457.9763
**897.9172**	449.4623	**8**	**L**	**7**	**747.8871**	374.4473
995.0339	498.0206	**9**	**P**	**6**	**634.7277**	**317.8676**
1082.1121	541.5597	**10**	**S**	**5**	**537.6110**	269.3092
**1139.1640**	**570.0857**	**11**	**G**	**4**	**450.5328**	225.7701
**1270.3566**	**635.6820**	**12**	**M**	**3**	**393.4809**	197.2442
1357.4348	679.2211	**13**	**S**	**2**	**262.2883**	131.6479
		**14**	**R**	**1**	175.2101	88.1088
