# Supplementary material for: Proteomics studies confirm the presence of alternative protein isoforms on a large scale
Source: Genome Biol. 2008 Nov 18;9(11):R162. doi: 10.1186/gb-2008-9-11-r162 (PMC2614494; doi:10.1186/gb-2008-9-11-r162)
Supplement: Additional data file 4 — Fragment ion masses for the phosphopeptide GFGMS*HSLPSGMDTEFSFPSSSSR, which is unique to the Sex lethal isoforms CG18350-PG, CG18350-PH, CG18350-PO, CG18350-PC, CG18350-PJ, CG18350-PN, are shown in tabular form. Detected ions are highlighted in red. [file gb-2008-9-11-r162-S4.pdf]

b^{1+}	b^{2+}	#	AA	#	y^{1+}	y^{2+}
58.0593	29.5333	**1**	**G**	**24**		
205.2359	103.1216	**2**	**F**	**23**	2559.6755	1280.3415
262.2878	131.6476	**3**	**G**	**22**	2412.4989	**1206.7532**
393.4804	197.2439	**4**	**M**	**21**	2355.447	**1178.2272**
560.5385	280.7729	**5**	**S[167]**	**20**	2224.2544	**1112.6309**
697.6796	349.3435	**6**	**H**	**19**	2057.1963	1029.1019
784.7578	392.8826	**7**	**S**	**18**	1920.0552	960.5313
**897.9172**	449.4623	**8**	**L**	**17**	**1832.977**	916.9922
995.0339	498.0206	**9**	**P**	**16**	**1719.8176**	860.4125
1082.1121	541.5597	**10**	**S**	**15**	1622.7009	811.8542
1139.164	570.0857	**11**	**G**	**14**	**1535.6227**	**768.3151**
**1270.3566**	635.682	**12**	**M**	**13**	**1478.5708**	739.7891
**1385.4432**	693.2253	**13**	**D**	**12**	**1347.3782**	674.1928
1486.5483	743.7778	**14**	**T**	**11**	**1232.2916**	**616.6495**
**1615.6638**	808.3356	**15**	**E**	**10**	**1131.1865**	566.097
**1762.8404**	881.9239	**16**	**F**	**9**	**1002.071**	501.5392
1849.9186	925.463	**17**	**S**	**8**	**854.8944**	427.9509
**1997.0952**	999.0513	**18**	**F**	**7**	**767.8162**	384.4118
2094.2119	1047.6096	**19**	**P**	**6**	**620.6396**	310.8235
2181.2901	1091.1487	**20**	**S**	**5**	523.5229	262.2652
2268.3683	1134.6878	**21**	**S**	**4**	436.4447	218.7261
2355.4465	**1178.2269**	**22**	**S**	**3**	349.3665	175.187
2442.5247	1221.766	**23**	**S**	**2**	262.2883	131.6479
		**24**	**R**	**1**	175.2101	88.1088
